# Association between serum galectin-3 and chronic obstructive pulmonary disease: A meta-analysis

**DOI:** 10.17305/bb.2024.10527

**Published:** 2024-12-01

**Authors:** Xiangyu Zhao, Bo Han, Wentao Tang, Shanshan Ji, Lie Wang, Jinbao Huang, Yizhong Hu, Jie Li

**Affiliations:** 1 Department of Clinical Laboratory, The First Affiliated Hospital of Anhui Medical University, Hefei, China; 2 Department of Clinical Laboratory, The People’s Hospital of Chizhou, Chizhou, China

**Keywords:** Chronic obstructive pulmonary disease, galectin-3, acute exacerbation, biomarker

## Abstract

Chronic obstructive pulmonary disease (COPD) is a significant public health issue characterized by progressive and irreversible airflow limitation. The aim of this meta-analysis was to determine the association between changes in serum galectin-3 levels and COPD and to assess the relationship between serum galectin-3 levels and acute exacerbations of COPD (AECOPD). Relevant observational studies were retrieved from electronic databases, including PubMed, Web of Science, Embase, Wanfang, and China National Knowledge Infrastructure (CNKI). A random-effects model was used to combine the data, incorporating the influence of between-study heterogeneity. Twelve case-control studies were included. The pooled results showed a significantly higher serum level of galectin-3 in patients with COPD compared to controls (standardized mean difference [SMD] 0.60; 95% confidence interval [CI] 0.40–0.80; *P* < 0.001; *I*^2^ ═ 68%). Further meta-analysis suggested higher levels of serum galectin-3 in patients with AECOPD compared to those with stable COPD (SMD 0.33; 95% CI 0.20–0.46; *P* < 0.001; *I*^2^ ═ 0%). Subgroup analyses according to the mean age of the participants, the proportion of males, and study quality scores did not significantly change the results (*P* for subgroup differences all > 0.05). In conclusion, patients with COPD were found to have higher serum levels of galectin-3, with levels further elevated in patients with AECOPD compared to those with stable COPD.

## Introduction

Chronic obstructive pulmonary disease (COPD) is a significant public health issue, often characterized by progressive and irreversible limitation of airflow [1–3]. The persistent airflow restriction is primarily due to bronchiolitis causing irreversible obstruction, but it can also be exacerbated by the destruction of lung tissue (emphysema) and the excessive production of mucus (chronic bronchitis) [4]. Patients with COPD experience repeated episodes of significantly increased symptoms (acute exacerbations of COPD [AECOPD]), involving more pronounced local and systemic inflammation. This leads to temporary deterioration in lung function, reduced quality of life, hospitalization, and an increased risk of further disease progression [5, 6]. Due to the complex nature of COPD’s pathophysiology, there is increasing interest in identifying potential biomarkers that can assist in both diagnosing and managing this condition [7, 8].

Galectin-3, a protein that binds to β-galactosides, has become a promising candidate due to its role in inflammation, fibrosis, tissue remodeling, and immune function [9–11]. An initial study involving patients with severe COPD indicated an elevated expression of galectin-3 and the accumulation of neutrophils in the small airway epithelium [12]. This was found to be linked to epithelial proliferation and airway obstruction [12]. A subsequent preclinical investigation suggested that exposure to cigarette smoke might trigger the release of galectin-3 in cultured airway epithelial cells, potentially contributing to the development of COPD [13]. However, previous studies examining changes in serum levels of galectin-3 among COPD patients have yielded conflicting findings [14–25]. While some studies reported higher serum levels of galectin-3 compared to healthy controls [18, 20, 23–25], others did not observe this difference [14, 17, 21, 22]. Given these uncertainties, we aim to investigate the link between serum levels of galectin-3 and COPD through a meta-analysis.

## Materials and methods

The Preferred Reporting Items for Systematic Reviews and Meta-Analyses (PRISMA) statement (2020) [26, 27] was followed in this study. The Cochrane Handbook [28] for systematic review and meta-analysis was referenced throughout the study. The study has been registered in the Open Science Framework with the registration number 10.17605/OSF.IO/WYCP7.

### Search strategy

Five electronic databases, including PubMed, Web of Science, Embase, Wanfang, and China National Knowledge Infrastructure (CNKI), were used for the literature search with a predefined combined search term: (1) “chronic obstructive pulmonary disease” OR “COPD” OR “chronic obstructive lung disease” OR “chronic obstructive airway disease” OR “emphysema” OR “chronic airflow limitation” OR “chronic airway obstruction” and (2) “galectin-3” OR “galectin 3”. The search syntax used in the meta-analysis is shown in Figure S1. Only studies with human subjects and published in English or Chinese peer-reviewed journals were included. A second-round check-up of the references of the relevant articles was also conducted. The final database search was completed on January 25, 2024.

### Inclusion and exclusion criteria

The inclusion criteria were as follows: (1) Observational studies in full-length articles; (2) Studies including adult patients with a confirmed diagnosis of COPD without other concomitant cardiopulmonary diseases, such as coronary artery disease, heart failure, or asthma, regardless of the disease status of COPD (AECOPD or stable COPD); (3) Studies measuring serum galectin-3 levels and comparing them between patients with COPD and healthy controls, or between patients with AECOPD and stable COPD; and (4) Studies where the difference in serum galectin-3 and its corresponding 95% confidence interval (CI) was reported or could be calculated from the original reports.

The exclusion criteria were as follows: (1) Reviews and meta-analyses; (2) Studies including patients with COPD and other concomitant cardiopulmonary diseases; (3) Studies measuring galectin-3 levels in bronchoalveolar lavage fluid; and (4) Studies comparing serum galectin-3 levels between patients with COPD and patients with other cardiopulmonary diseases. For studies with potentially overlapping patient populations, the one with the largest sample size was included in the meta-analysis.

### Data collection and quality assessment

Two independent authors conducted the literature search and analysis, data collection, and study quality assessment separately. If discrepancies were encountered, the corresponding author participated in the discussion for final judgment. Data on study information, study design, diagnosis, demographic factors of the studied population, proportion of current smokers in the studied population, methods for measuring serum galectin-3, and variables that were adjusted or matched between cases and controls were extracted. Study quality assessment was achieved via the Newcastle–Ottawa Scale (NOS) [29], with scoring based on criteria for participant selection, comparability of the groups, and the validity of the outcomes. The scale ranged between 1–9 stars, with a larger number of stars indicating higher study quality.

### Ethical statement

Ethical approval was not required for this study in accordance with local/national guidelines. Written informed consent to participate in the study was not required in accordance with local/national guidelines.

### Statistical analysis

The primary outcome of the meta-analysis was to investigate the difference in serum galectin-3 between patients with COPD and healthy controls, while the secondary outcome was to compare serum galectin-3 between patients with AECOPD and stable COPD. The difference in serum galectin-3 between groups was summarized as the standardized mean difference (SMD) and 95% CI because different methods were used for measuring galectin-3 [28]. Between-study heterogeneity was estimated with the Cochrane *Q* test and the *I*^2^ statistic [30, 31], with *I*^2^ > 50% reflecting significant statistical heterogeneity. A random-effects model was applied to combine the results by incorporating the influence of statistical heterogeneity [28]. Sensitivity analysis, by excluding one study at a time, was used to evaluate the robustness of the findings [28]. For analyses with significant statistical heterogeneity, a univariate meta-regression analysis was performed to evaluate the potential impact of study characteristics in continuous variables on the results, such as mean age, percentage of males, and NOS of the included studies. Additionally, subgroup analysis was performed to evaluate the impact of study characteristics on the results, such as disease status, mean age, proportion of males, and NOS, with the medians of the continuous variables as cutoff values for defining subgroups. By constructing funnel plots, publication bias was estimated based on the visual judgment of the symmetry of the plots, supplemented with Egger’s regression asymmetry test [32]. A *P* < 0.05 was considered statistically significant. The RevMan (Version 5.1; Cochrane Collaboration, Oxford, UK) and Stata (version 17.0; Stata Corporation, College Station, TX, USA) software packages were used for these analyses.

## Results

### Study inclusion

**Figure 1.**
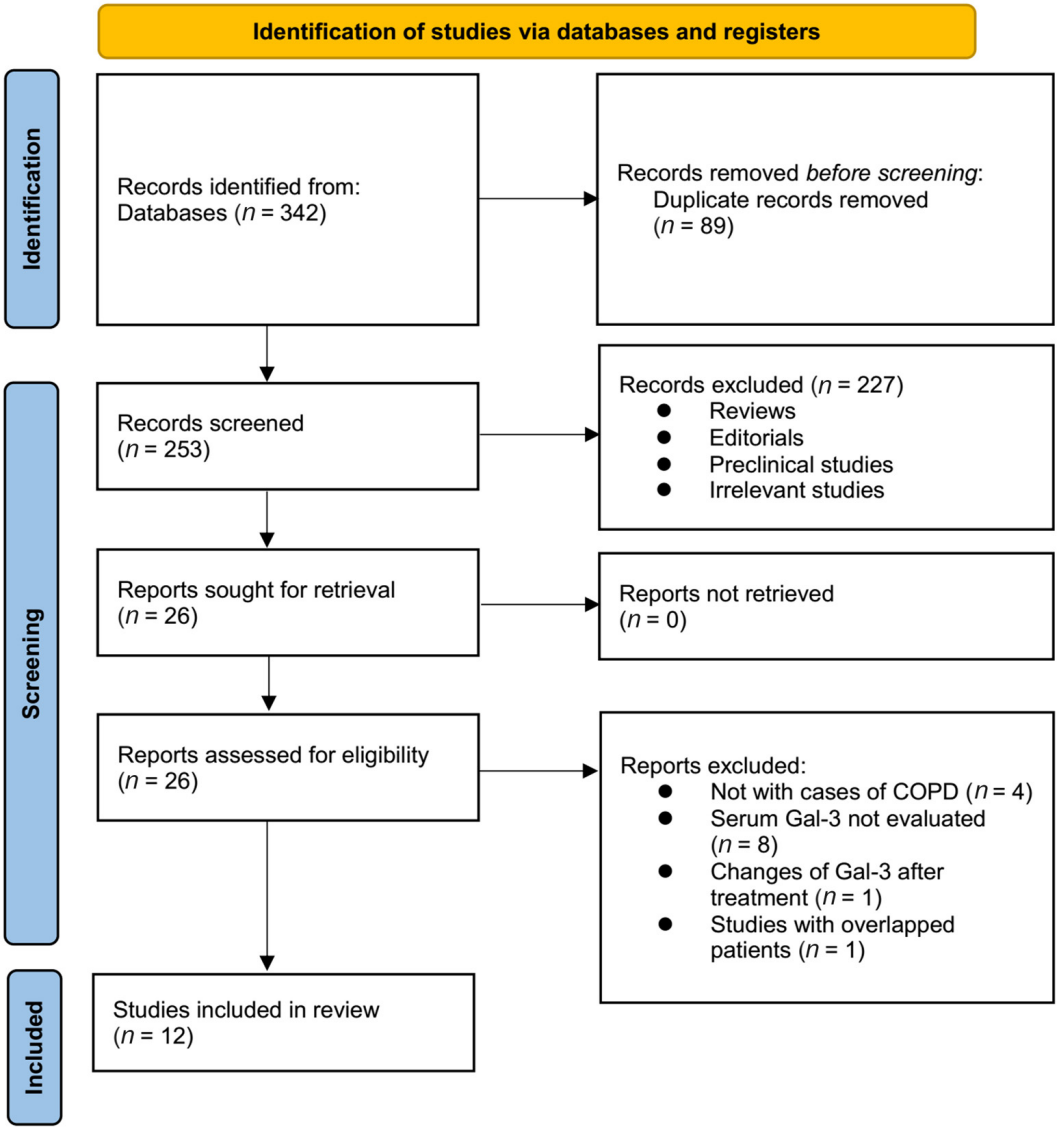
**Flowchart illustrating the process of literature search and study identification.** COPD: Chronic obstructive pulmonary disease; Gal-3: Galectin-3.

The process for identifying relevant studies for inclusion in the meta-analysis is presented in Figure 1. In brief, 342 potentially relevant records were obtained after comprehensive searches of the three databases, and 89 of them were excluded due to duplication. Subsequently, a screening considering the titles and abstracts of the remaining records further led to the exclusion of 227 more studies, mostly because they were not related to the aim of the meta-analysis. Accordingly, the full texts of the 26 remaining records were read by two independent authors, and 14 of them were further removed for various reasons, as listed in Figure 1. Finally, 12 observational studies remained and were considered suitable for the subsequent quantitative analyses [14–25].

### Overview of the study characteristics

Table 1 presents the summarized characteristics of the included studies. Overall, 12 case-control studies involving 1167 patients with COPD and 573 healthy controls were included in the meta-analysis [14–25]. These studies were published between 2015 and 2024 and were performed in Austria, The Netherlands, China, and Sweden. The mean ages of the included population ranged from 47.7 to 69.2 years, with the percentages of males ranging from 37.9% to 77.5%. Serum galectin-3 was measured with the chemiluminescent microparticle immunoassay (CMIA) in one study [14] and with the enzyme-linked immunosorbent assay (ELISA) in the others [15–25]. Potential variables such as age, sex, body mass index (BMI), and smoking status were matched or adjusted to varying degrees in ten studies [15–21, 23–25]. The NOS of the included studies ranged from six to nine stars, suggesting overall moderate to good study quality (Table 2).

**Table 1:** Characteristics of the included studies

**Study**	**Country**	**Design**	**No. of patients with AECOPD**	**No. of patients with stable COPD**	**No. of healthy controls**	**Mean age (years)**	**Males (%)**	**Current smoking (%)**	**Methods for measuring serum Gal-3**	**Variables matched or adjusted**
Mueller, 2015	Austria	CC	15	0	22	48.1	70.3	32.4	CMIA	None
Pouwels, 2015	The Netherlands	CC	40	40	0	63.6	77.5	45	ELISA	Age, sex, smoking, and BMI
Feng, 2017	China	CC	44	44	0	69.2	77.3	20.5	ELISA	Age, sex, smoking, and BMI
Shen, 2018	China	CC	0	100	100	55.2	79	NR	ELISA	Age, sex, and smoking
Liu, 2019	China	CC	60	60	0	68.7	76.7	63.3	ELISA	Age, sex, BMI, and smoking
Li, 2019	China	CC	0	42	30	47.7	50	NR	ELISA	Age and sex
Du, 2020	China	CC	151	107	129	68.9	57.9	NR	ELISA	Age and sex
Mao, 2020	China	CC	40	40	40	56.7	60	NR	ELISA	Age and sex
Sundqvist, 2021	Sweden	CC	0	56	20	61.1	37.9	82.1	ELISA	None
Wang, 2021	China	CC	71	79	74	58	59.5	NR	ELISA	Age and sex
Wang, 2023	China	CC	0	64	60	64.2	66.9	NR	ELISA	Age, sex, and BMI
Zhang, 2024	China	CC	60	54	98	56.2	61.3	NR	ELISA	Age, sex, and BMI

COPD: Chronic obstructive pulmonary disease; AECOPD: Acute exacerbation of COPD; Gal-3: Galectin-3; CMIA: Chemiluminescent microparticle immunoassay; ELISA: Enzyme-linked immunosorbent assay; CC: Case-control; BMI: Body mass index; NR: Not reported.

**Table 2:** Study quality evaluation via the Newcastle–Ottawa Scale

**Study**	**Adequate definition of the cases**	**Representativeness of the cases**	**Selection of controls**	**Definition of controls**	**Controlled for age and sex**	**Controlled for other confoundings**	**Ascertainment of the exposure**	**Same method of ascertainment of exposure for cases and controls**	**Non-response rate**	**Overall**
Mueller, 2015	1	0	1	1	0	0	1	1	1	6
Pouwels, 2015	1	0	1	1	1	1	1	1	1	8
Feng, 2017	1	0	1	1	1	1	1	1	1	8
Shen, 2018	0	0	1	1	1	1	1	1	1	7
Liu, 2019	1	1	1	1	1	1	1	1	1	9
Li, 2019	1	0	1	1	1	0	1	1	1	7
Du, 2020	0	1	1	1	1	0	1	1	1	7
Mao, 2020	1	1	1	1	1	0	1	1	1	8
Sundqvist, 2021	1	0	1	1	0	0	1	1	1	6
Wang, 2021	1	0	1	1	1	0	1	1	1	7
Wang, 2023	1	0	1	1	1	1	1	1	1	8
Zhang, 2024	1	1	1	1	1	1	1	1	1	9

### Serum galectin-3 levels between patients with COPD and healthy controls

**Figure 2.**
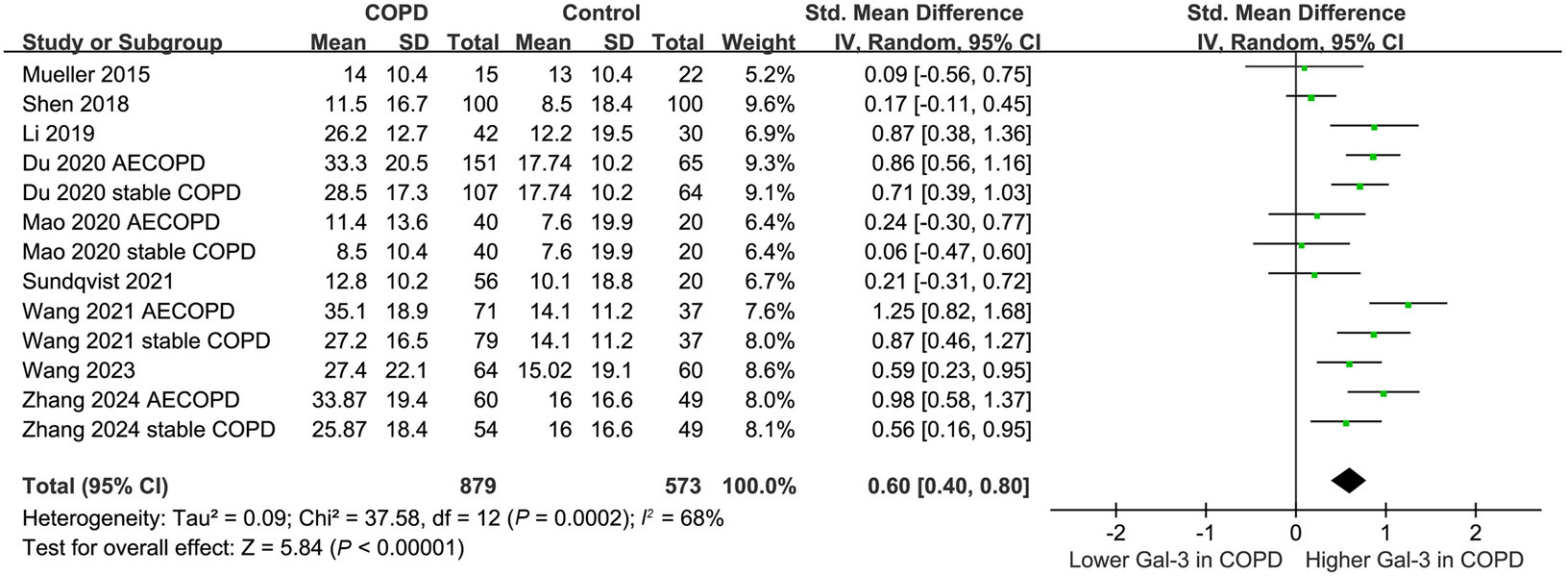
**Forest plots for the meta-analysis comparing the serum galectin-3 levels between patients with COPD and healthy controls.** COPD: Chronic obstructive pulmonary disease; AECOPD: Acute exacerbation of COPD; SD: Standard deviation; CI: Confidence interval; Gal-3: Galectin-3.

Nine studies compared serum levels of galectin-3 between patients with COPD and healthy controls [14, 17, 18, 20–25]. Since four of them reported the difference of galectin-3 between cases and controls according to the disease status of COPD (AECOPD or stable COPD) [20, 21, 23, 25], these datasets were included independently, and the sample sizes of the control groups were equally split to avoid unit-of-analysis errors as detailed in the Cochrane Handbook [28]. Overall, the pooled results showed a higher serum level of galectin-3 in patients with COPD compared to healthy controls (SMD ═ 0.60, 95% CI 0.40–0.80; *P* < 0.001; *I*^2^ ═ 68%; Figure 2).

Subsequent sensitivity analysis by excluding one dataset at a time showed consistent results (SMD ═ 0.55–0.65; *P* all < 0.05).

The meta-regression analysis suggested that study characteristics, such as mean age, percentage of males, and NOS did not significantly affect the results (Table 3).

**Table 3:** Univariate meta-regression analysis for the SMD of serum galectin-3 between patients with COPD and healthy controls

**Variables**	**SMD of serum Gal-3**		
	**Coefficient**	**95% CI**	***P* values**
Mean age (years)	0.013	−0.025–0.050	0.48
Males (%)	−0.0088	−0.0343–0.0168	0.47
NOS	0.062	−0.204–0.327	0.62

SMD: Standardized mean difference; COPD: Chronic obstructive pulmonary disease; Gal-3: Galectin-3; CI: Confidence interval; NOS: Newcastle–Ottawa Scale.

**Figure 3.**
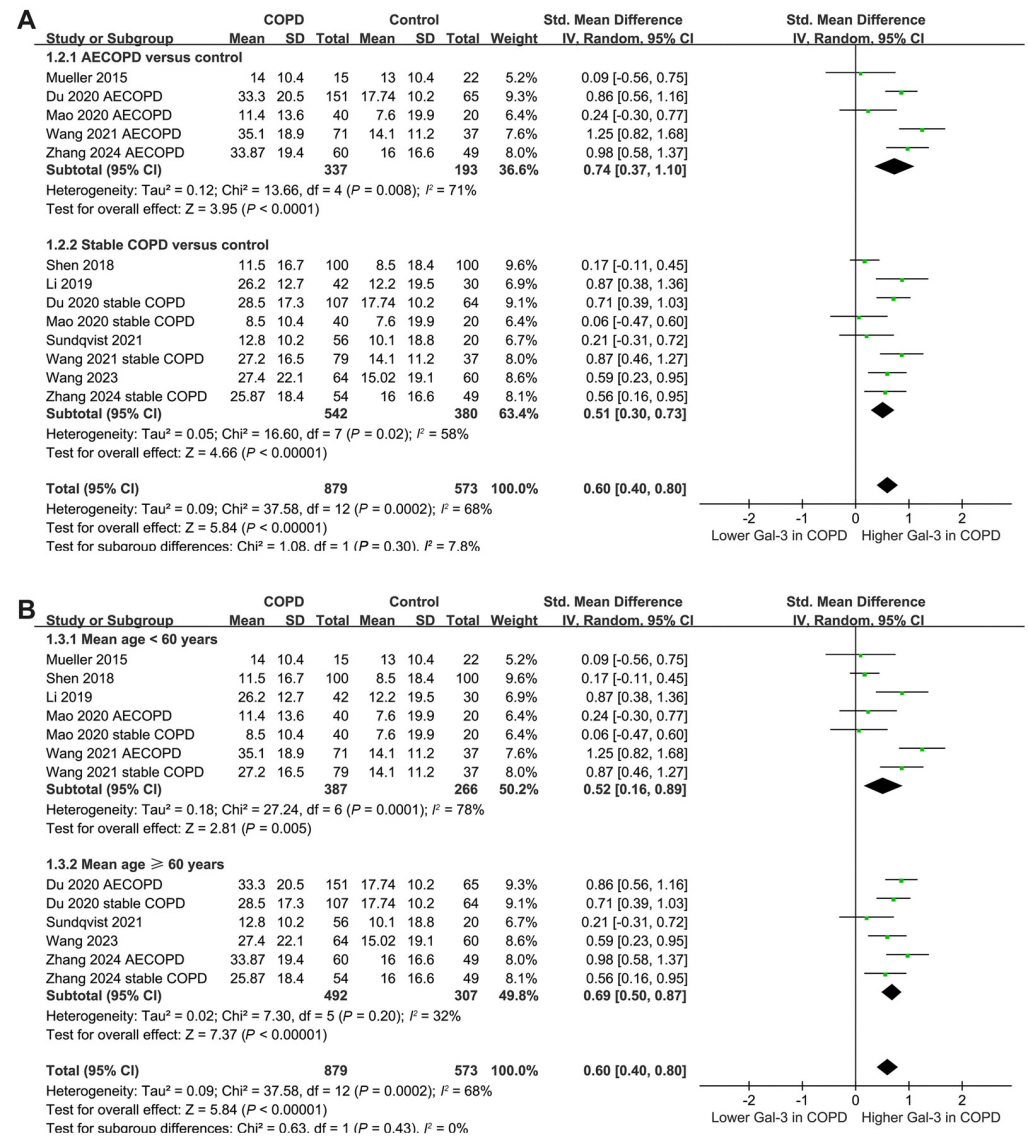
**Forest plots for the subgroup analyses comparing the serum galectin-3 levels between patients with COPD and healthy controls.** (A) Forest plot illustrating the subgroup analysis based on the disease status; (B) Forest plot illustrating the subgroup analysis based on the mean age of the population. COPD: Chronic obstructive pulmonary disease; AECOPD: Acute exacerbation of COPD; SD: Standard deviation; CI: Confidence interval; Gal-3: Galectin-3.

**Figure 4.**
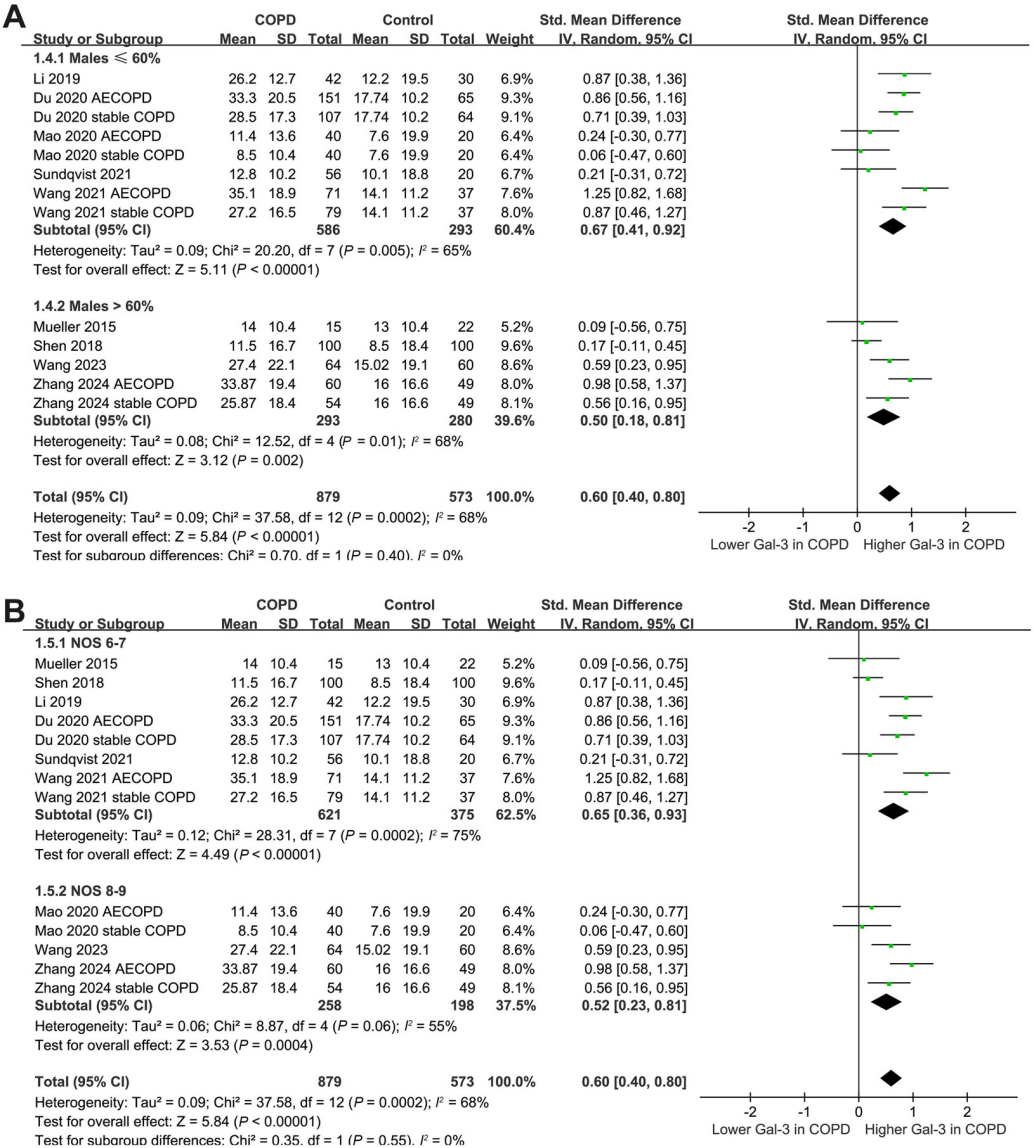
**Forest plots for the subgroup analyses comparing the serum galectin-3 levels between patients with COPD and healthy controls**. (A) Forest plot illustrating the subgroup analyses based on the proportion of males; (B) Forest plot illustrating the subgroup analyses based on the study quality scores. COPD: Chronic obstructive pulmonary disease; AECOPD: Acute exacerbation of COPD; SD: Standard deviation; CI: Confidence interval; Gal-3: Galectin-3.

Results of the subgroup analysis based on the disease status and mean age of the patients revealed that these variables did not significantly affect the findings (*P* for subgroup difference ═ 0.30 and 0.43, respectively; Figure 3A and 3B). Similar findings were observed for studies with the proportions of males ≤ or > 60% (*P* for subgroup difference ═ 0.40; Figure 4A). Additionally, the subgroup analysis based on the quality scores of the included studies revealed that this variable did not significantly affect the findings (*P* for subgroup difference ═ 0.55; Figure 4B).

### Serum galectin-3 between patients with AECOPD and stable COPD

**Figure 5.**
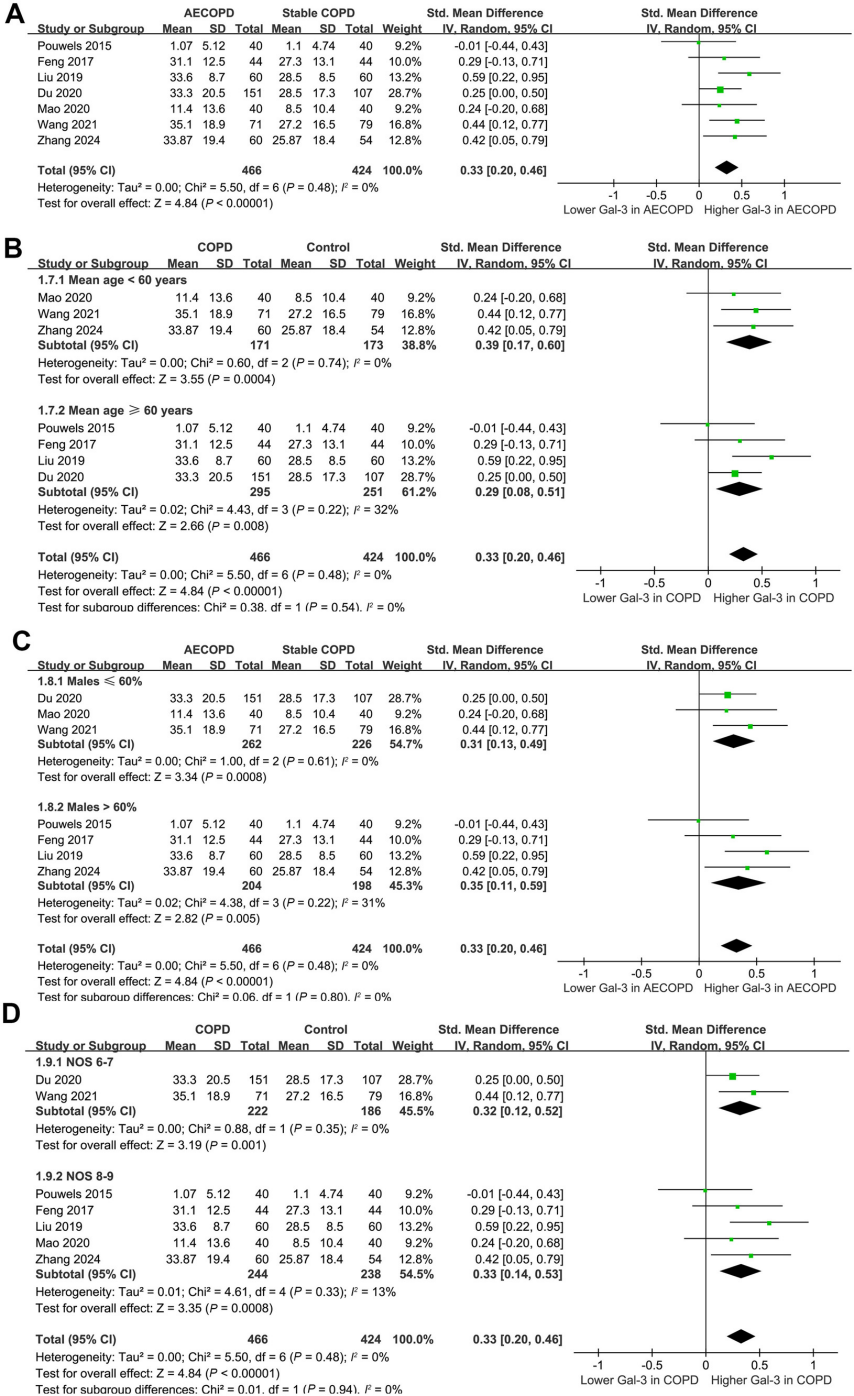
**Forest plots for the meta-analysis comparing the serum galectin-3 level between patients with AECOPD and stable COPD.** (A) Forest plot illustrating the overall meta-analysis; (B) Forest plot illustrating the subgroup analyses based on the mean ages of the participants; (C) Forest plot illustrating the subgroup analyses based on the proportions of males; (D) Forest plot illustrating the subgroup analyses based on the study quality scores. COPD: Chronic obstructive pulmonary disease; AECOPD: Acute exacerbation of COPD; SD: Standard deviation; CI: Confidence interval; Gal-3: Galectin-3.

The meta-analysis of seven studies [15, 16, 19–21, 23, 25] further suggested a higher level of serum galectin-3 in patients with AECOPD compared to stable COPD (SMD ═ 0.33, 95% CI 0.20–0.46; *P* < 0.001; *I*^2^ ═ 0%; Figure 5A). Sensitivity analysis by omitting one study at a time did not significantly affect the results (SMD ═ 0.29–0.36; *P* all < 0.05). Further exploring meta-analysis suggested similar results in patients with mean ages < and ≥ 60 years, in studies with the proportions of males ≤ or > 60%, and in studies with different quality scores (*P* for subgroup difference all > 0.05; Figure 5B–5D).

### Publication bias evaluation

**Figure 6.**
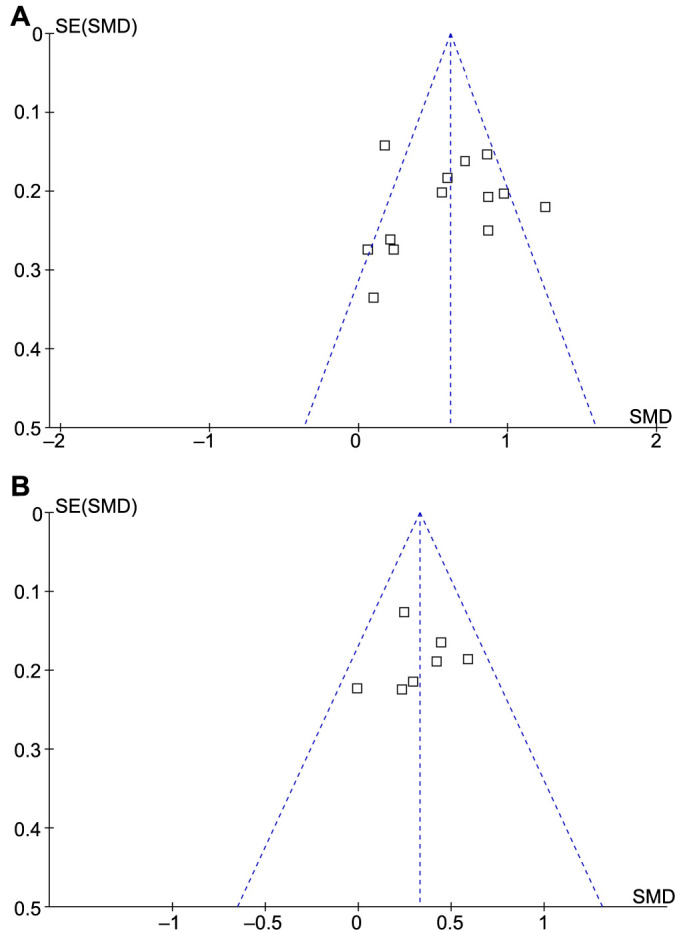
**Funnel plots for the publication biases underlying the meta-analyses.** (A) Funnel plot illustrating the meta-analysis comparing the serum galectin-3 level between patients with COPD and healthy controls; (B) Funnel plot illustrating the meta-analysis comparing the serum galectin-3 level between patients with AECOPD and stable COPD. COPD: Chronic obstructive pulmonary disease; AECOPD: Acute exacerbation of COPD; SMD: Standardized mean difference.

The funnel plots for the meta-analyses of the difference in serum galectin-3 between patients with COPD and healthy controls, and between patients with AECOPD and stable COPD are shown in Figure 6A and 6B. The symmetrical nature of the funnel plots suggested a low likelihood of publication biases. Results of Egger’s regression test also showed low risks of publication biases underlying the meta-analyses (*P* ═ 0.91 and 0.78, respectively).

## Discussion

This meta-analysis synthesized the findings from 12 case-control studies and found that individuals with COPD had higher levels of galectin-3 in their serum compared to healthy controls. Furthermore, it was noted that individuals experiencing AECOPD also had elevated levels of galectin-3 compared to those with stable COPD. Subsequent subgroup analyses according to age, sex, and study quality scores showed similar results. These findings indicate that increased serum galectin-3 levels could serve as a potential biomarker for both chronic and acute states of COPD.

This research may represent the first meta-analysis to compile data on the changes in serum galectin-3 levels among COPD patients. Before interpreting the results, it is important to acknowledge the rigorous methodology applied in this meta-analysis. A comprehensive search of five widely used electronic databases yielded 12 recent observational studies relevant to this meta-analysis’s objectives. Moreover, only studies involving COPD patients without other concurrent cardiopulmonary conditions were considered, aiming to minimize potential confounding effects from comorbidities on the meta-analysis results. Additionally, various sensitivity and subgroup analyses confirmed the robustness of the primary findings and indicated that neither individual datasets nor study characteristics, such as mean age, percentage of males, or study quality scores significantly influenced the outcomes. Overall, these results highlight the potential utility of serum galectin-3 as a marker for identifying COPD and AECOPD patients, particularly among current smokers.

The potential reasons for the connection between elevated galectin-3 and COPD are complex. One study revealed increased galectin-3 expression in the small airway epithelium of COPD patients, along with an accumulation of neutrophils, which may contribute to epithelial growth and airway blockage in these individuals [12]. Another study found that exposure to cigarette smoke extract notably raised galectin-3 gene expression in airway epithelial cells from COPD patients but not those from healthy controls [13]. The induction of galectin-3 following cigarette smoke exposure was associated with neutrophilic airway inflammation [13]. Lastly, a recent study suggested that the build-up of galectin-3 in bronchial epithelial cells isolated from COPD patients could indicate insufficient autophagic breakdown and accelerated cellular aging—both known mechanisms underlying COPD progression [33]. Further research is necessary to uncover the main molecular pathways responsible for the link between increased galectin-3 levels and COPD.

Overall, the meta-analysis on serum galectin-3 levels in COPD reveals its potential as a diagnostic biomarker and prognostic indicator, with elevated levels associated with disease presence and exacerbations. Clinically, galectin-3 could aid in early COPD detection and stratification of patients based on exacerbation risk, and serve as a therapeutic target. Future research should focus on longitudinal studies to validate these associations, mechanistic investigations to understand its role in COPD pathophysiology, clinical trials to evaluate targeted interventions, and subgroup analyses to identify responsive patient groups. Overall, galectin-3 shows promise in improving COPD management by providing insights into disease mechanisms and guiding personalized treatment strategies.

This study also has some limitations that should be noted. One significant issue is that this meta-analysis focused on comparing the different serum levels of galectin-3 between cases and controls, and it did not determine the optimal cutoff value of galectin-3 for discriminating COPD and AECOPD from the patient population. Additionally, all included studies were case-control studies with a cross-sectional design. Therefore, evaluating the dynamic changes of serum galectin-3 during COPD exacerbation and following treatments is necessary. Moreover, other potential confounding factors may affect the association between serum galectin-3 and COPD. For example, statins have been suggested to influence serum galectin-3 levels, which in turn may affect the association between galectin-3 and COPD [34]. Lastly, our meta-analysis solely included observational studies, thus precluding the establishment of a causal relationship between galectin-3 in the development and acute exacerbation of COPD.

## Conclusion

The results of the meta-analysis indicate that patients with COPD had higher serum galectin-3 levels. Moreover, individuals with AECOPD exhibited elevated galectin-3 compared to those with stable COPD. While further prospective studies are required to validate the connection between increased serum galectin-3 and the onset and acute exacerbation of COPD, this meta-analysis supports the potential utility of serum galectin-3 as a biomarker for identifying patients with COPD and AECOPD.

## Supplemental data

**Figure S1.**
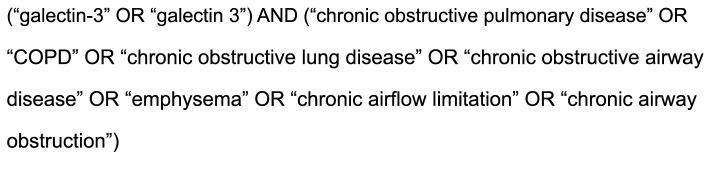
**The search syntax used in the meta-analysis.** COPD: Chronic obstructive pulmonary disease.

## Data Availability

All the data generated during the study is included within the manuscript.
